# Spatial patterns and policy implications of invasive flora in the Horn of Africa

**DOI:** 10.1038/s41598-026-54640-8

**Published:** 2026-05-28

**Authors:** Kflay Gebrehiwot, Magdalena Szymura

**Affiliations:** 1 Institute of Agroecology and Plant Production, Wrocław University of Environmental and Life Sciences, Grunwaldzki Sq. 24A, 50-363 Wrocław, Poland; 2 Department of Biology, College of Natural and Computational Sciences, Samara University, P. O. Box 132, Semera, Ethiopia

**Keywords:** Alien plants, Assessment protocols, Naturalized alien species, Non-native, Transboundary collaboration, Ecology, Ecology, Plant sciences

## Abstract

Invasive plants are fast-spreading species that pose serious and often irreversible threats to native biodiversity. This study presents a regional-scale analysis of the elevational distribution of invasive plant species, their relationship with road proximity, and their presence across different land uses. Data were synthesized following the Reporting Standards for Systematic Evidence Syntheses protocol using multiple databases, including Web of Science, Scopus, and Google Scholar. A total of 250 invasive species distributed in 63 families were documented. The dominant families were Fabaceae (16.8%), Asteraceae (9.6%) and Poaceae (6.4%). Herb was the predominant growth form, accounting for 47.6%, while liana/shrub was the least represented at just 1.2%. The native range of the species were predominantly Neotropical (30%) and African (23.1%). About 5.1% of the species were either helophytes or aquatic. Furthermore, nine (3.6%) of them were either hemiparasites or holoparasites. Kenya and Ethiopia had the highest records of invasive plant species, while Djibouti and Eritrea had the lowest. Invasive species declined significantly with increasing elevation (*P* < 0.001) and were declining further from roads (*P* < 0.008). Most occurrences were recorded in cultivated areas (33.51%), shrublands (23.1%), and urban or built-up regions (16.66%). The study highlights four main factors: (1) lack of reported protocols for assessing impact and classifying invasiveness, (2) potential ‘snowball effect’ in reporting, (3) paucity of data on invasive species and (4) lack of proper definition of operational terms. As a result, the number of invasive species reported in this study may not accurately represent the true extent in the region. To address this, it is recommended to develop or adopt region-specific, expert-led assessment tools and establish a collaborative framework for managing invasive plant species.

**Supplementary Information:**

The online version contains supplementary material available at 10.1038/s41598-026-54640-8.

## Introduction

Invasive plants are species that spread quickly and cause significant harm to native biodiversity, disrupt local ecosystems, and diminish the valuable services nature provides to people^1^. Their introduction and spread can have complex, often irreversible effects on native plant species, communities, and ecosystem functioning. Beyond ecological damage, biological invasions also result in substantial economic losses and adverse impacts on human well-being and health. For instance, in 2019 alone, the annual economic cost of addressing the negative impacts of invasive species was estimated to be approximately US$423 billion globally^1^.

Over the past few decades, their impact has grown at an alarming rate worldwide. More than four percent of the vascular plants (16,429) in the world are reported to be naturalized alien plant species^2,3^ and about seven percent (1061) of them are invasive^1^. This trend is closely linked to the global increase in invasive alien species^1–3^ and it is expected that climate change would aggravate it. Projections also suggest that the number of invasive plant species will continue to rise across most biogeographic regions in the coming two decades^4,5^.

Africa faces a particularly severe invasive species problem, with invasive alien plant species reported to be adversely affecting livelihoods in more than 70% of African countries^6^. Despite commitments under the UN Convention on Biological Diversity’s Aichi Target 9 to control or eliminate priority invasive alien species by 2020, little progress has been recorded in many African countries^6,7^. Major barriers include constrained financial resources, lack of legal frameworks, insufficient operational systems, and limited capacity at ports of entry to detect invasive species^6^. There is also a noted lack of regional coordination in management approaches across cross-country borders, even for species that occur across neighboring countries^8^. For instance, *Neltuma juliflora* (Fabaceae) is reported to be an invasive species in all of the Horn of African countries (study area). However, the authors could not find any collaborative strategic efforts to manage the invasive species across the region. South Africa stands out as the only country in Africa with a reasonably comprehensive and accurate list of invasive species across all types, having conducted research on invasive plant species for much longer than other African nations^9^. The management practices are also by far better there than other African countries^6^.

In almost five decades, only 45 invasive plant species caused an estimated economic cost of US$8.6 billion in Africa. Of these, the *Acacia* species alone imposed an estimated economic cost of US$3.4 billion^10^. Given the limited scope of cost estimates for many invasive plant species and the steadily increasing number of invasions across the continent, the actual economic impact is likely much higher than currently reported. For example, the study by Diagne et al.^10^ estimated the economic costs for three species, *Neltuma juliflora*, *Parthenium hysterophorus* (Asteraceae), and *Opuntia stricta* (Cactaceae), and only in two countries, Ethiopia and Kenya, within the present study area. Furthermore, due to weak quarantine regulations in many African countries, the introduction of invasive species continues to rise^6,7,11^.

Except for some invasive alien plant species, in many parts of the continent, research on introduction pathways of the invasive alien plant species remains limited^11^. Developed countries tend to experience repeated introductions of closely related alien plant groups through established trade networks^12^. In contrast, developing regions often manifest more diverse phylogenetic patterns, reflecting various introduction pathways^12^.

For example, an extremely notorious weed, *Parthenium hysterophorus,* is suspected to have been accidentally introduced to Ethiopia in the mid-1980s from contaminated imported grains through trail and track corridors^13^. Although *P. hysterophorus* was first recorded in small coffee plantations in Kenya in the mid-1970s^14^, the most aggressive invasion was reported three decades later, which was suspected that *P. hysterophorus* population was introduced from Ethiopia^15^. The construction of road networks and the nature of the species aggravated the invasion of *P. hysterophorus* into neighbouring countries. Another notorious invasive alien species in the Horn of Africa is *N. juliflora*, introduced to the region in the 1970s and 1980s for environmental rehabilitation due to its drought tolerance^16^. However, it has since caused numerous unforeseen damaging social, economic, and ecological effects, threatening agropastoral communities’ subsistence and food security by occupying rangelands, creating impenetrable thickets, reducing diversity and abundance of native species diversity, and even causing animal health issues^8^. *Neltuma juliflora* has been spreading in desert and semi-desert ecosystems in all countries of the study area. If all the suitable habitats for *N. juliflora* are invaded by the species, the economic cost could reach up to USD7 billion in the coming three decades in East Africa^17^. Due to the massive and irreversible damages of the invasive plant species, there is an urgent need for more comprehensive economic studies to inform management decisions, otherwise the consequences could be devastating^18^.

Although invasive plant species occur on nearly all continents, their impacts are generally less severe in developed countries due to technological advancement and stronger economic capacity, while developing countries experience more pronounced effects^19^. In developing countries such as the Horn of Africa plant invasions pose significant challenges, often exacerbated by the existing low-income. Additive effects of multiple global change factors on plant invasions are common, indicating that as global environmental changes persist, the risk of plant invasions continues to rise^20^.

Despite the extremely limited information on the introduction pathways of invasive species to the study area, there are several factors that facilitate the introduction and success of invasive alien plant species. The distribution of invasive plant species is significantly shaped by a complex interplay of anthropogenic activities and environmental factors such as road density and elevation^12,21,22^. Roads are identified as primary pathways and crucial conduits for the introduction, spread, and establishment of invasive plants in various habitats, including nature reserves and mountain ecosystems^23,24^. They usually facilitate the dispersal of propagules through different transport mechanisms, as well as by animals using roadways as travel paths^24^. Furthermore, the construction and maintenance of roads create disturbed habitats characterized by altered soil conditions, increased light availability, and reduced native vegetation cover, which are highly favorable for neophytes, ruderals, invasive and potentially invasive species to establish and thrive by making resources available and lessening competition from native species^25^. Studies consistently show that the richness and density of invasive species are negatively correlated with road proximity^24,26^. Beyond just the physical road, other anthropogenic factors like proximity to human settlements, tourist activity, and land-use changes also act as significant drivers for plant invasions due to the disturbances they induce on the ecosystems.

Regarding elevation, invasive plant species richness generally exhibits a consistent pattern of decreasing with increasing elevation^21,27,28^. This decline is largely attributed to the increasingly harsh climatic conditions encountered at higher altitudes, such as lower temperatures, and shorter growing seasons, which act as environmental filters limiting species establishment and survival. This phenomenon is often explained by directional ecological filtering, where non-native species are typically introduced at lower elevations due to higher anthropogenic propagule pressure, and only those with broad climatic tolerances or pre-adaptations are able to successfully spread upwards^27,29,30^. Additionally, human activities and associated disturbances, which are major drivers of invasion, tend to decrease with rising elevation, further contributing to the lower prevalence of invasive species in high-altitude environments^23^. However, with ongoing climate change and ever-increasing human population, this premise might not remain the same because a significant number of species are shifting their range allowing some invasive species to colonize previously uninvaded high elevation areas. The gap in effective policy for invasive species management might also serve to aggravate the situation^19^.

Studies on invasive plant species in the Horn of Africa have largely been species-specific and geographically local^8^. Research indicates that the region is significantly underrepresented in invasive species studies^1,19,31,32^. Therefore, this study seeks to conduct a comprehensive regional-level analysis of the elevational distribution patterns of invasive plant species, their correlation with road proximity, and their distribution across various land uses. By addressing the current research gap at this broader scale, the study will generate empirical evidence to support the need for regional collaboration in developing strategic invasive plant species management plans. The present study would motivate researchers to delve into transboundary and transdisciplinary investigations on invasive alien plant species across the Horn of Africa and beyond.

## Materials and methods

### Study area

**Figure 1:**
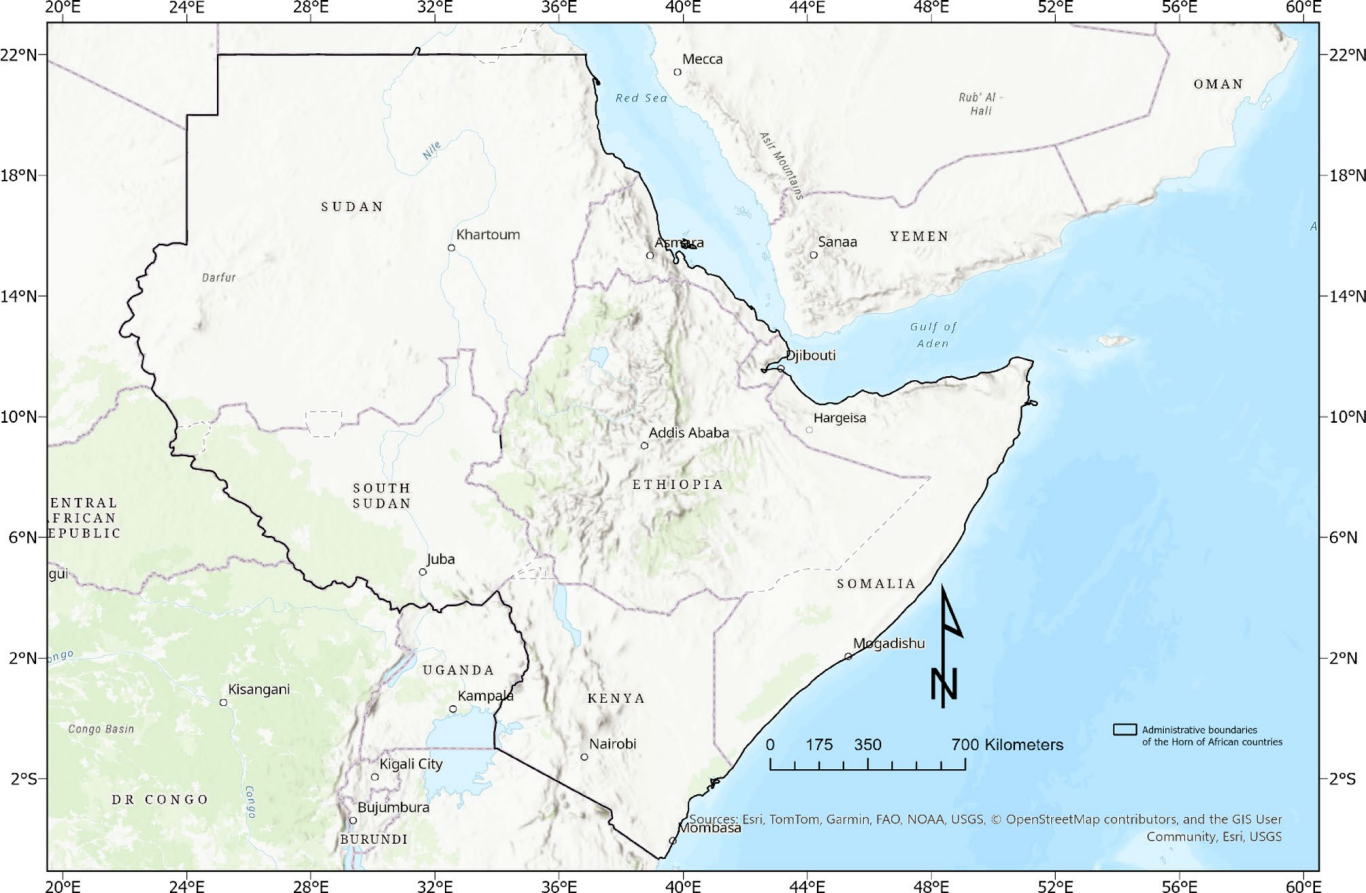
Map of the study area—Horn of Africa (Somali Peninsula). Basemap source: Esri, Tom Tom, Garmin, FAO, NOAA, USGS OpenStreetMap contributors, and the GIS User Community, Esri, USGS. The map was created using ArcGIS Pro 3.4.

The study area encompasses six countries in the Horn of Africa, specifically Djibouti, Eritrea, Ethiopia, Kenya, Somalia, and Sudan (Fig. 1). The area covers about 5 million square kilometers. This region is sometimes referred to as the Somali Peninsula, although this term typically excludes countries like Kenya and Sudan. Geographically, it is situated in Eastern Africa.

The Horn of Africa features a diverse climate shaped by its varied geography. Coastal and lowland areas in Somalia, Djibouti, and Eritrea are typically warm and arid, while lowlands in Ethiopia and Kenya experience warm, semi-arid conditions. In contrast, the high mountains of Ethiopia and Kenya are cool and humid, the highlands are warm and humid, and western Kenya is characterized by hot, humid weather^33^. The region’s climate is highly sensitive to variability due to its geographic position and the influence of several factors, including the Intertropical Convergence Zone (ITCZ), the Sahara Desert, particularly in the northeast, El Niño Southern Oscillation (ENSO), and fluctuations in Indian ocean currents and surface temperatures^34,35^. Climate change is expected to further alter conditions in the coming decades^36^. The region’s wide range of elevations contributes to significant spatial variation and ecological heterogeneity^37^, supporting a high diversity of plant species. Land cover data show the landscape is predominantly composed of grasslands and shrublands, interspersed with small patches of remnant forests^38^.

Despite its unique flora with high level of endemism and ecological significance, the region is critical and hosts three globally recognized biodiversity hotspots^39,40^. These are the Horn of Africa biodiversity hotspot, Eastern Afromontane Hotspot, and Coastal Forests of Eastern Africa. The Horn of Africa biodiversity hotspot is characterized by extreme aridity and habitat degradation; it predominantly covers the lowland areas of Djibouti, Eritrea, Ethiopia, and Somalia. Pastoralism and agropastoralism is the most common livelihood in this biodiversity hotspot. The Eastern Afromontane Hotspot, on the other hand, is characterized by high altitude ecosystems and the forests of the region are dominantly found in this area. In the study area, this hotspot covers the montane forests and highlands of Ethiopia and Kenya. For several reasons, this biodiversity hotspot is the most populated area and agriculture is the predominant livelihood. The third biodiversity hotspot, Coastal Forests of Eastern Africa, mainly covers the southeastern margins of the study area. It is mainly associated with the coastal regions of Kenya and Tanzania. It is recognized for its high species turnover and unique flora^41^.

### Search strategy

Data were sourced from indexing databases, particularly Web of Science Core Collection (Topic), Scopus (Article title, Abstract and Keywords), and Google Scholar (allintitle). The following Boolean search string was employed: (("invasive plant*" OR "alien plant*" OR "non-native species" OR “weed” OR "exotic plant*") AND (“management” OR “control” OR “eradication” OR “restoration” OR “impact” OR “policy”) AND ("Horn of Africa" OR Ethiopia OR Eritrea OR Djibouti OR Somalia OR Sudan OR Kenya)). This strategy was designed to capture literature related to invasive plant species in the region, including their management practices and existing policy frameworks.

Additional invasive species data were gathered from specialized databases, including the CABI Invasive Species Compendium^42^, the Global Register of Introduced and Invasive Species^43^, GBIF^44^, the Global Naturalized Alien Flora database (GloNAF)^2,3^, RAINBIO^45,46^, Global Invasive and Alien Traits and Records (GIATAR)^47^. The datasets were downloaded from the listed websites and databases. The data were filtered by country name, and records classified as invasive, as well as duplicate entries, were excluded. Although grey literature was also reviewed, it did not reveal any additional or distinct invasive plant species.

**Figure 2:**
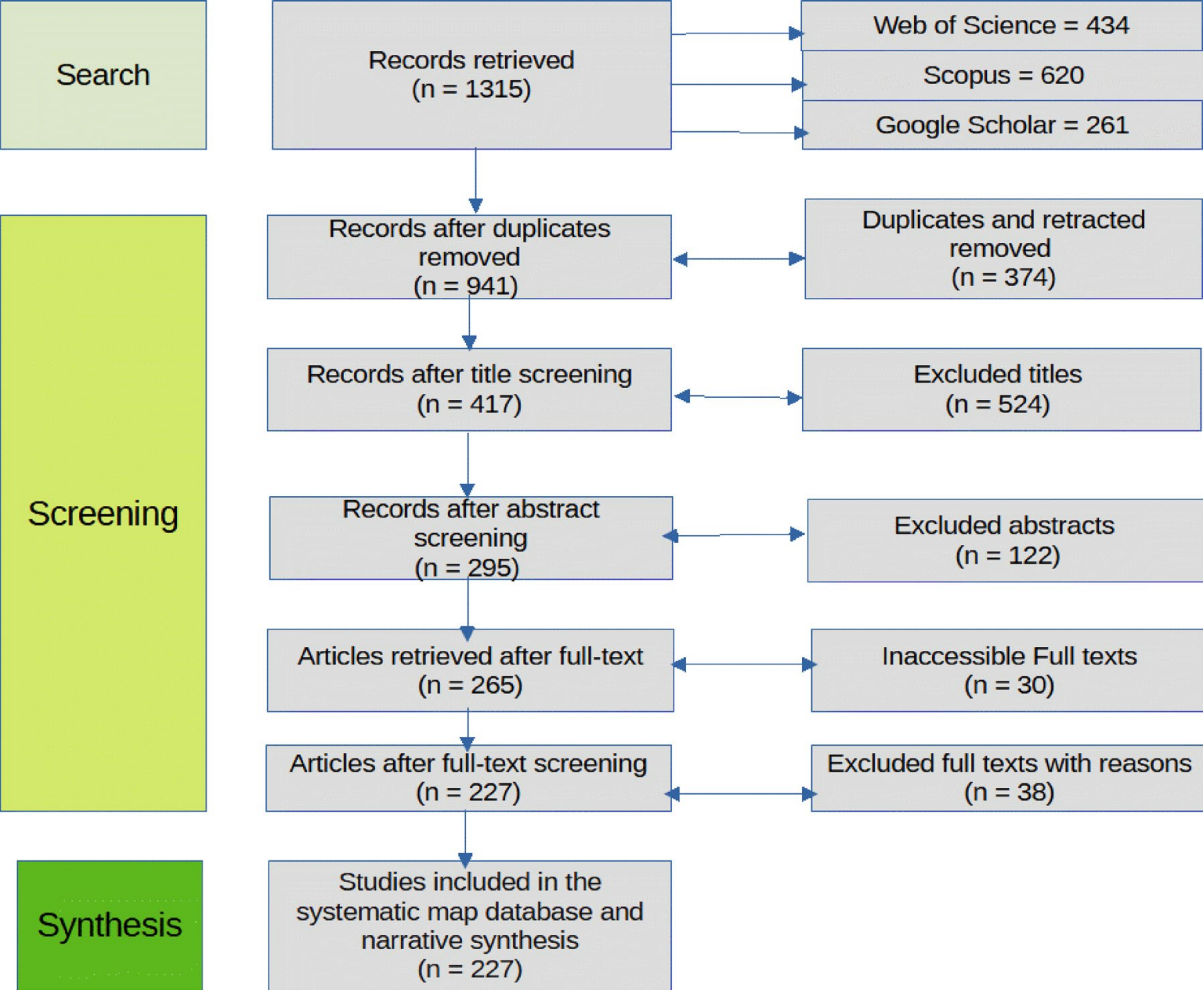
Schematic diagram of study search, screening and synthesis. This is a Reporting Standards for Systematic Evidence Syntheses (ROSES) protocol.

Following an iterative search process, a total of 1,315 studies were retrieved: 620 from Scopus, 434 from Web of Science, and 261 from Google Scholar. The study adhered to the Reporting Standards for Systematic Evidence Syntheses (ROSES) protocol^48^ (Fig. 2).

### Inclusion–exclusion criteria and data screening

The literature search was conducted without language restrictions. Almost all of the retrieved studies were published in English, with the exception of one article in Arabic, which was excluded due to the authors’ inability to read Arabic and the lack of a translation budget. Several studies were also missing full-text access. To address this, emails requesting full texts were sent to corresponding authors; however, only five articles and one book were successfully obtained. As a result, studies without accessible full texts were excluded.

Additionally, articles published in predatory journals^49^ or those considered below academic standards (based on the authors’ judgement) were excluded although few were considered for a justifiable reason. Only studies that recorded or mentioned invasive plant species were selected for the review. Our aim was to synthesize existing research on invasive plant species in the region; therefore, we did not provide our own judgment on species invasiveness. Instead, we analyzed and summarized classifications reported by other studies, noting contradictions and methodological flaws in the discussion section. Species with uncertain invasiveness were marked with an asterisk or hashtag (* or #) in Supplementary Tables 2. The dataset included both terrestrial and aquatic invasive plant species. Species information from these studies was incorporated into the invasive species dataset for the Horn of Africa. In total, 227 studies (listed in Supplementary Table 1) were included to extract data on invasive plant species in the region.

### Geographic coordinates of the invasive plant species

Latitude and longitude data for the invasive plant species were obtained from published studies and databases, including GBIF^44^. GBIF data were filtered by scientific name, country, and location (including coordinates), with duplicates removed. After data cleaning, a total of 37,635 occurrence points (136 points per species on average) were used for the spatial analysis. Approximately 17% of species have either no geographic coordinate records or fewer than five coordinate points. The geographic coordinates were used for four main purposes: (1) to analyze the distribution of invasive species, including growth forms, along an elevation gradient, (2) to assess their relationship with road proximity, (3) to map their occurrences within the study area, and (4) to assess the distribution of invasive alien species across different land cover types. The elevation in the study area ranges from below 100 m below sea level to over 4300 m above sea level.

### Data analysis

Descriptive statistics, a Generalized Linear Model (GLM), and Sørensen’s similarity coefficient were employed to analyze the data. A GLM was used to examine both the relationship between elevation and invasive plant species distribution. Since the occurrence data were not collected from fixed sample plots or sites, elevation was categorized into eight ranges, and species richness and frequencies were aggregated accordingly. The elevation classes are supposed to represent the different vegetation types in the Horn of Africa. To analyze the distance from the road and richness correlation, we first calculated the point distance from the road using the Near tool in ArcGIS Pro 3.4.0. Then, Poisson regression analysis was applied to analyze the road distance–invasive species occurrence correlation. Over dispersion was also checked using DHARMa R package^50^. To address overdispersion identified in the initial analysis, we employed a Negative Binomial (glm.nb) linear model in R. Subsequent diagnostic checks confirmed the model fit, yielding a dispersion ratio of 0.97, indicating that the overdispersion was successfully mitigated.

To assess the similarity of invasive plant species among the six Horn of Africa countries, Sørensen’s similarity coefficient was calculated using the Bray method based on species presence/absence data across the six target countries.

Additionally, the native ranges of invasive plant species were analyzed descriptively. The biogeographic realms defined in the most recent global floristic map by Liu et al.^51^ was used to determine species’ native regions. To determine the native biogeographic range and ensure taxonomic standardization, the Plants of the World Online (POWO) database (https://powo.science.kew.org/) was consulted. In addition, native range information for invasive plant species was retrieved using the rWCVP package (version 1.3.0)^52^, while taxonomic standardization was performed with the WorldFlora R package^52^.

The Africa land cover map at 100 m spatial resolution was obtained from Copernicus Land Service: Global Land Cover/Land Use^53^. The land cover map was clipped by the study area. Then, the occurrence points were overplayed over the land cover map to extract the value points. Accordingly, the occurrence records in each land cover class were analyzed.

## Results

### Composition, taxonomic diversity and growth form

The present study recorded 250 invasive plant species in the Horn of Africa (Djibouti, Eritrea, Ethiopia, Kenya, Somalia and Sudan) (Supplementary Table 2). About 25% of the species are native but recorded as invasive within the study area.

**Figure 3:**
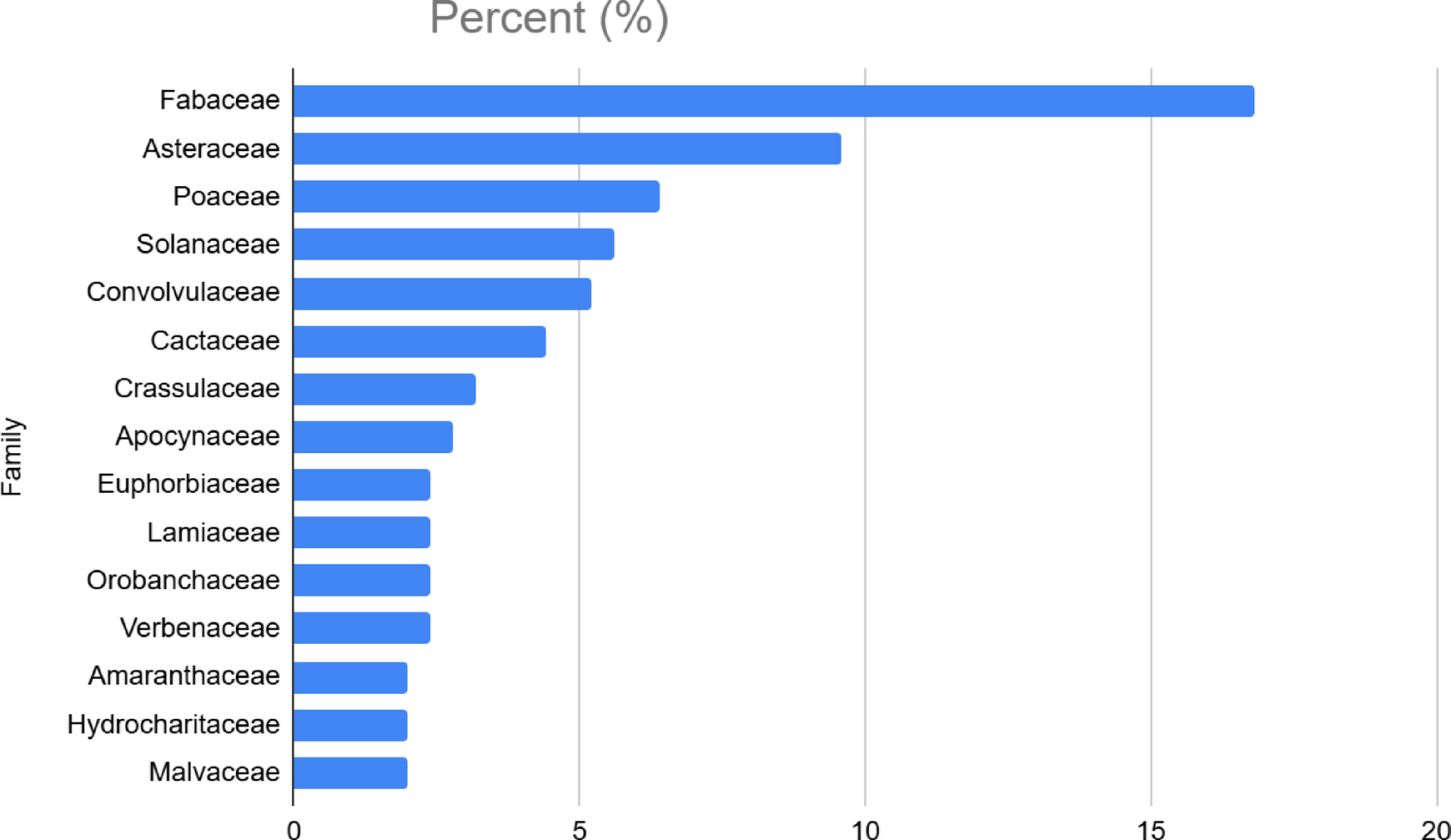
Invasive plant families represented by more than five species.

These are distributed in 63 families (Fig. 3). The dominant families are Fabaceae (16.8%), Asteraceae (9.6%) and Poaceae (6.4%). Kenya (204, 81.6%) and Ethiopia (113, 45.20%) were found to record the highest invasive plant species whereas the lowest species were recorded in Djibouti (4, 1.6%) and Eritrea (9, 3.6%). About 5.1% of the species were found to be either Helophytes (semiaquatic) or aquatic. Furthermore, nine (3.6%) of the invasive plant species reported were either hemiparasites or holoparasites. These species belong to the Orobanchaceae and Convolvulaceae family. Although the majority of reported invasive plants (66.8%) were found in only one of the Horn of Africa countries included in the study, approximately 25.2% were shared between two countries, and 7.2% were present in three to five countries. Notably, two species, *N. juliflora* and *Leucaena leucocephala* (Fabaceae), were reported as invasive in all six target countries, accounting for 0.80% of the total.

**Figure 4:**
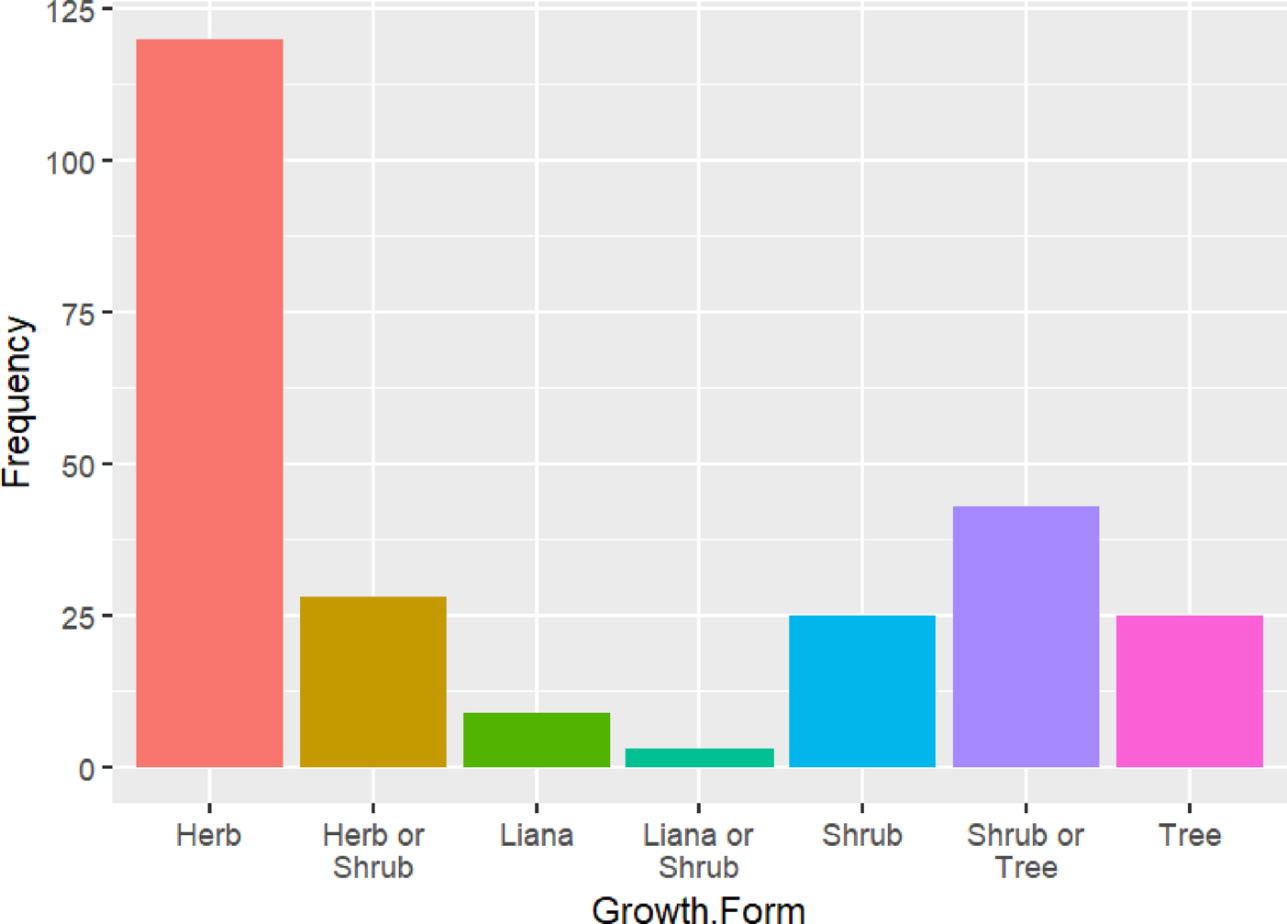
Growth form of the invasive plant species.

The growth form is predominated by herbs (47.6%), shrubs or small trees (16.8%), and herb/shrub (11.2%). Shrub/Liana (SL) were represented by the least number of invasive species (Fig. 4).

**Figure 5:**
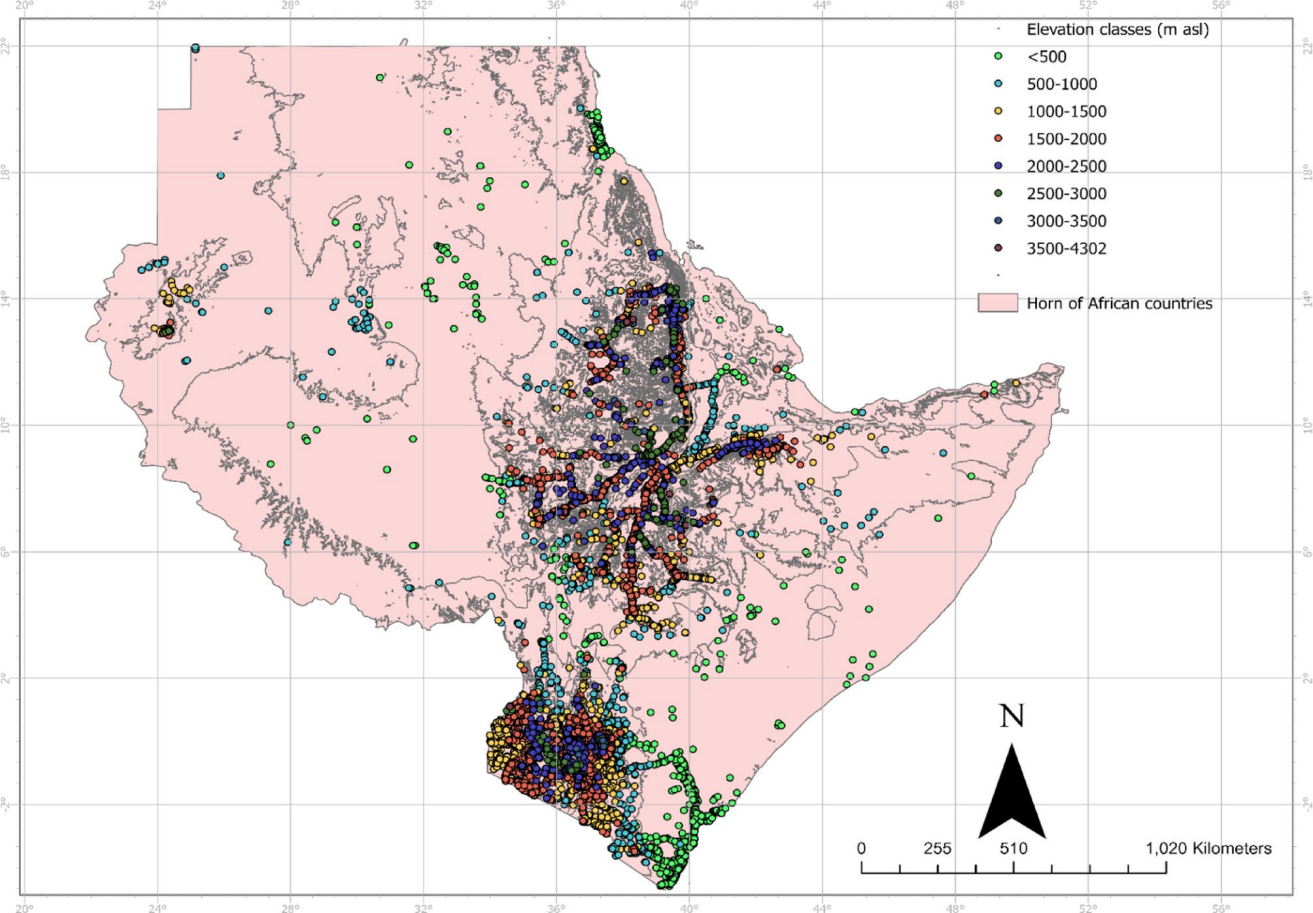
The geographic coordinates (spatial distribution) of the invasive species in the Horn of Africa. The geographic coordinates used to produce this map were obtained from GBIF^44^ and published articles. The map was created using ArcGIS Pro 3.4.

The map of invasive plant species occurrence points (Fig. 5) shows that these species are distributed throughout the Horn of Africa, with notably higher records in Ethiopia and Kenya. In contrast, despite Sudan’s large size, it has very few recorded occurrences.

### Sørensen’s dissimilarity index

The analysis of similarity among Horn of Africa countries showed that the highest Sørensen’s similarity index (1-dissimilarity coefficient) was between Eritrea and Somalia (57%), followed by Ethiopia and Kenya (50%). The lowest similarity was observed between Djibouti and Kenya, at just 5%, followed by Djibouti and Ethiopia, which is only 8% (Table 1).

**Table 1:**
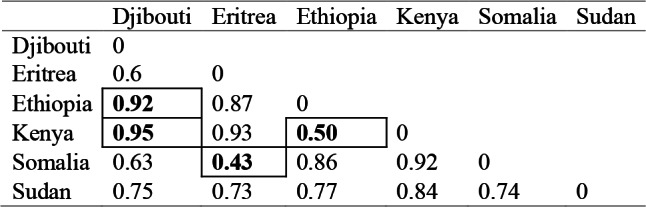
Sørensen’s dissimilarity matrix table.

### Origin of the invasive plants

**Figure 6:**
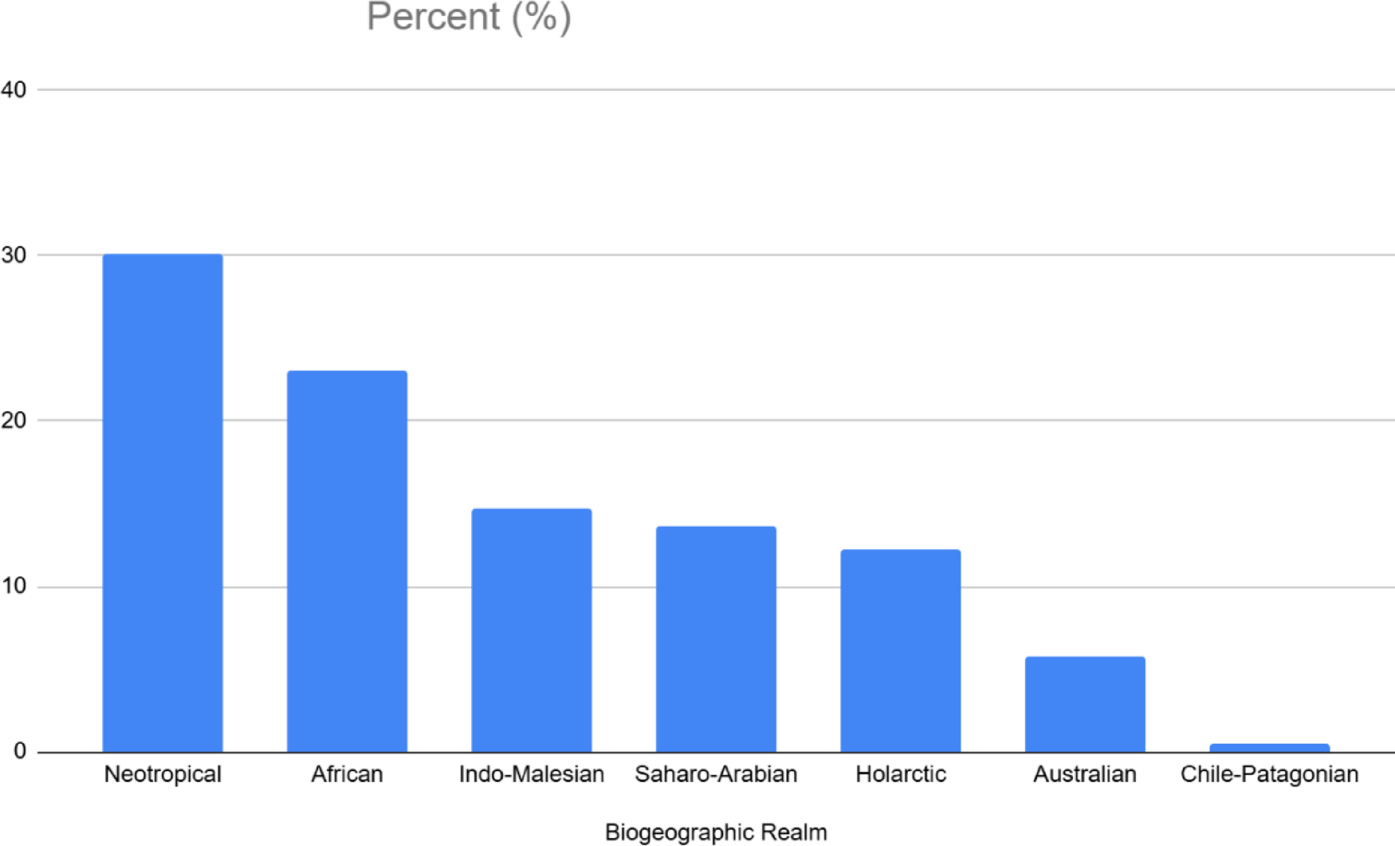
Origin of the invasive species from biogeographic realms perspectives.

The study found that the majority of invasive plant species originated from the Neotropical biogeographic realm (30%), followed by the African (also known as Afrotropical) realm (23.1%). The Chile-Patagonian realm contributed the fewer, with less than 1% of the species (Fig. 6). Nevertheless, it must be noted that a species can have more than one native geographic range (for example, *Cyperus rotundus* has five native biogeographic realms namely African, Indo-Malesian, Saharo-Arabian, Australian, and Holarctic).

### Invasive species occurrences along an elevation gradient

**Figure 7:**
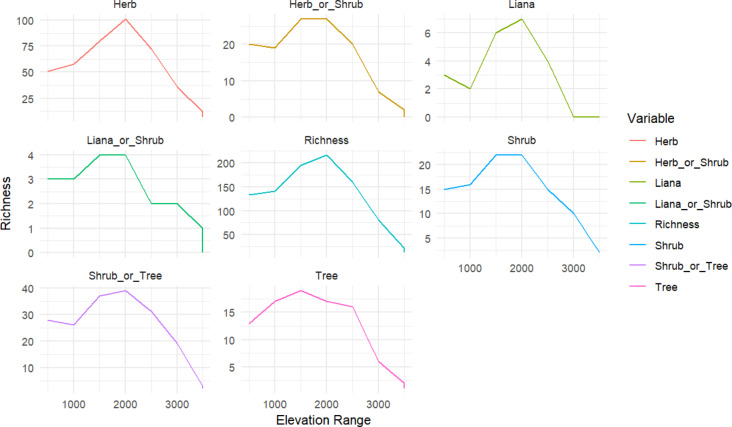
Invasive plant species growth form distribution pattern along an elevation gradient.

The distribution of invasive plant species along the elevation gradient exhibited a significant decline towards higher elevations (*P* < 0.001) (Fig. 7). The majority of invasive plants were concentrated at lower elevation areas, with peak occurrences between 1500 and 2000 m above sea level. In contrast, the highest elevation class, representing Afroalpine vegetation above 3500 m, recorded the fewest occurrences (48 points). A similar distribution pattern was observed across all growth forms. However, woody invasive species were entirely absent from the upper elevation class (Fig. 7).

### Road proximity and invasive plant occurrence

**Figure 8:**
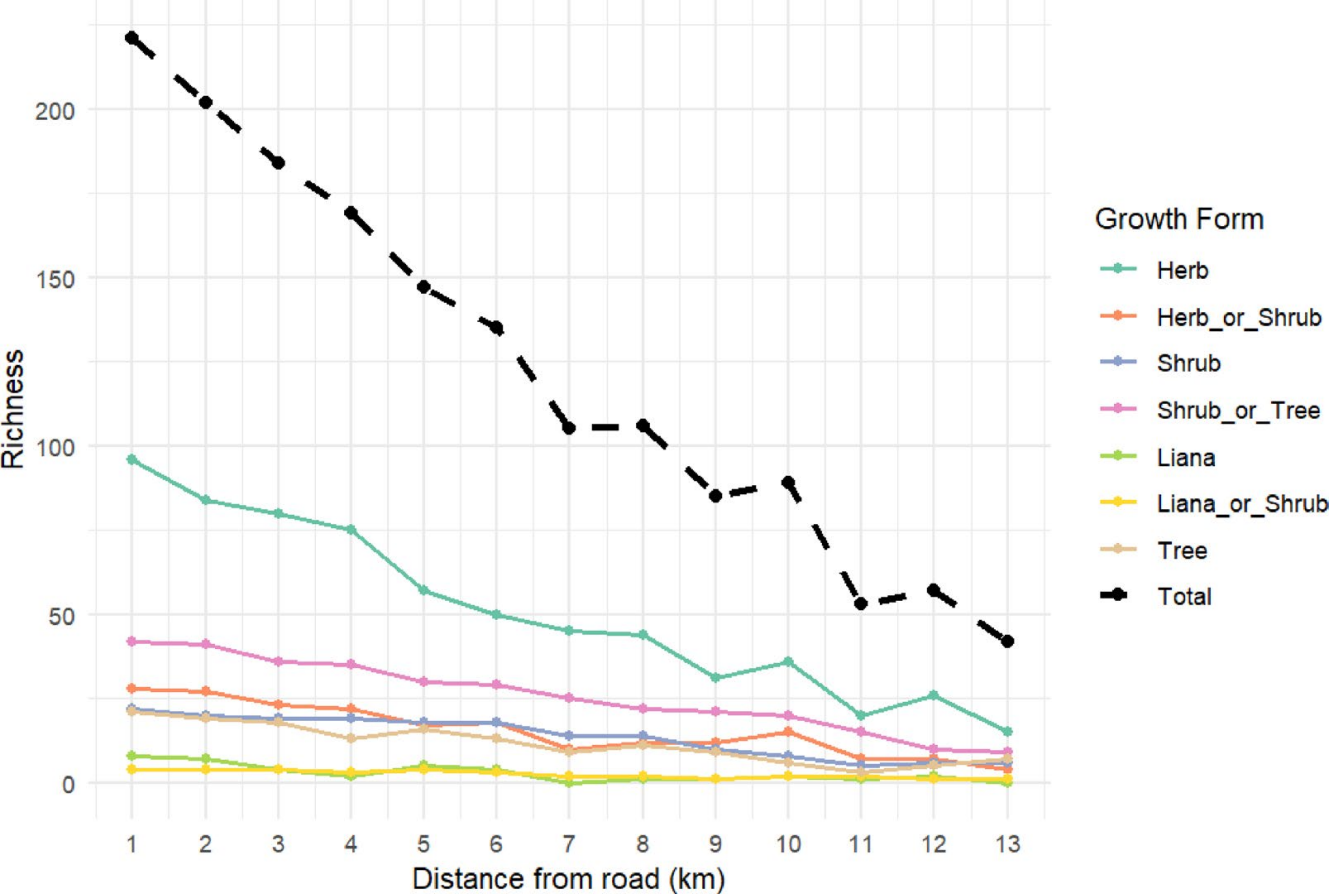
Invasive plant species richness pattern along road distance.

The road proximity of invasive plant occurrence points showed a significant (Adj. R-square = 0.54, *P* < 0.008) monotonic increase towards a short road distance (Fig. 8). About 70% of the occurrence points were recorded below 1 km distance from the road. The mean and standard deviation were 1.7and 3.36 respectively.

### Distribution of invasive plants across landcover types

**Figure 9:**
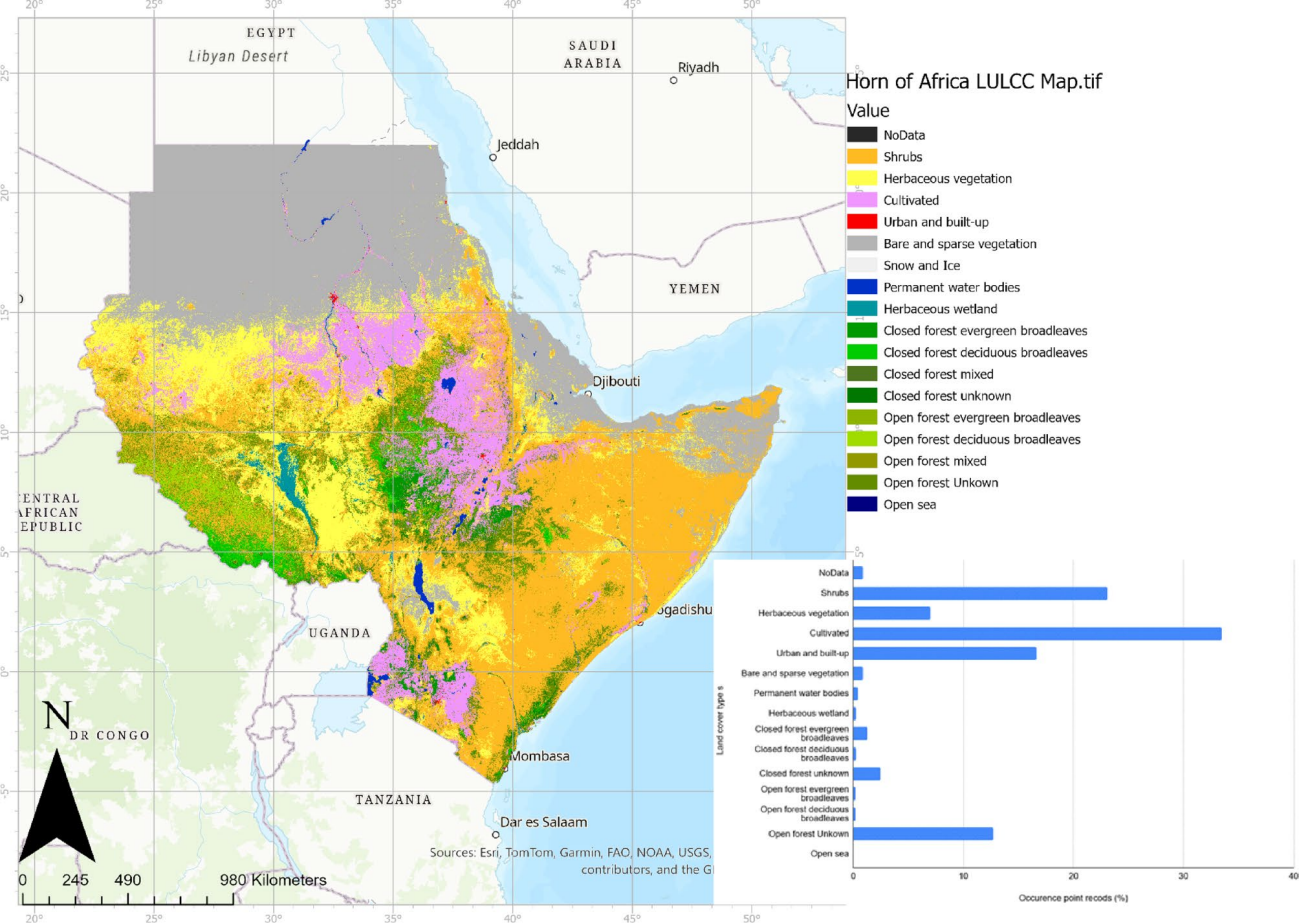
Distribution of invasive plant species across the land cover types in the Horn of Africa. The land cover map was obtained and re-produced from Buchhorn^53^. The map was created using ArcGIS Pro 3.4.

The highest number of occurrence records were observed in the cultivated (33.51%), shrubs (23.1%), and urban & built-up areas (16.66%). However, the lowest records were in open sea (0.02%), open forest evergreen broadleaves (0.23%), and open forest deciduous broadleaves (0.24%) (Fig. 9). When the aggregates are analyzed, cultivated land and urban and built-in areas comprised half of the invasive species recorded in all land cover types.

## Discussion

### Composition, taxonomic diversity and growth form

Two hundred fifty (250) invasive plant species have been documented in the study area. This comprises more than three-fold of the invasive plant species reported by Pyšek et al.^54^, which was 76, for the target countries in the study area. Although we argue that the number of invasive plant species reported in the article is significantly underestimated, Mungi et al.^55^ recently revealed that Eastern Africa, which includes the area covered by the present study, hosts the second-highest number of invasive plants, following Southern Africa. However, the number of invasive species reported in the present study may not precisely reflect the true extent of invasive species in the region for four key reasons.

First, there was no standardized assessment protocol reported or used to determine the invasive status of these species, highlighting the need for a national or regional evaluation framework. Some species labeled as invasive may not meet that classification based on actual field conditions. For example, *Jacaranda mimosifolia* (Bignoniaceae) (in Ethiopia and Kenya) and *Psidium guajava* (Myrtaceae) (in Ethiopia, Kenya, and Sudan) are reported as invasive. However, based on the first author’s field and research experience in Ethiopia, these species may not currently exhibit invasive behavior. Moreover, although *Tithonia diversifolia* (Asteraceae) was reported to be either rare or uncommon by Witt et al.^18^, it was reported as invasive in Ethiopia in some literature. This needs some sort of re-assessment. The fact that numerous of the invasive plant species recorded have either no geographic coordinates or fewer than five coordinate points highlights the need to assess the status of the reported invasive plant species. This discrepancy is likely due to the absence of a consistent evaluation method, either through adoption of existing tools like the Environmental Impact Classification for Alien Taxa (EICAT) or the development of region-specific assessment criteria^56,57^.

Secondly, some researchers seem to assume that a species invasive in one area will behave similarly elsewhere, which might not always be the case^58–60^. For example, if a study classifies a species as invasive without following a standardized assessment protocol, it is still likely to be cited by other studies. One such case is a review paper on invasive plants in Ethiopia^61^, published in a molecular biology journal by MedCrave, a publisher listed as predatory^49^. Despite this, the paper has received over 120 citations on Google Scholar. Many of the references it uses to support claims about species’ invasiveness are grey literature and do not disclose any formal classification approaches. Nevertheless, researchers in Ethiopia and elsewhere continue to cite such sources. This illustrates a ‘*snowball effect*’ in invasive plant research, particularly in parts of the Horn of Africa.

Third, large parts of the study area, particularly Sudan, Djibouti, Eritrea, and Somalia, are data-deficient. In countries like Sudan, where scientific literature is limited despite the country’s vast size, the actual number of invasive species may be underestimated^32^.

A fourth possible reason could be the inappropriate or inconsistent understanding of the biological invasion related terms such as invasive species, exotic/alien species, established/naturalized alien, or bush encroacher plants. The lack of clear and standardized definitions in many studies for these related concepts may lead to confusion about what actually constitutes an invasive species. This might either inflate or underestimate the extent of invasion. A recent study underscored that lack of standard invasive species-related terminologies has caused convoluted and inconsistent usage in invasion science, necessitating the proposal of a simplified and standardized framework^62^.

The predominance of Fabaceae, Asteraceae, and Poaceae is expected taking into account that these are the top species-rich families across the globe both in their native range and beyond. Numerous studies^54,63^ reported these families as the most invasive plants-rich families elsewhere in the world. Their success is mainly associated with the physiological mechanism to fix nitrogen (Fabaceae), diverse pollination and dispersal mechanisms, fast growth and adaptability to harsh environments^64–66^.

Despite the representation of all growth forms, herbaceous invasive species were by far predominant and woody species was the least represented. Herbs as dominant and woody species such as trees and liana as the least invasive plants were reported by numerous scholars elsewhere in the world^54,67,68^. The fast growth rate and several adaptation mechanisms to different environments enable the herbaceous plants to be more successful outside their native range^69^.

The highest similarity in invasive plant species was observed between Eritrea and Somalia, followed by Ethiopia and Kenya. This is likely due to their geographic proximity and similar climatic conditions, which contribute to comparable ecological characteristics across the region. For example, both Eritrea and Somalia are part of the Arid Horn biodiversity hotspot, which is characterized by low annual rainfall and high temperature. Additionally, the limited representation of Eritrea and Somalia in biological invasion literature may have also influenced the observed high similarity, as fewer records can lead to apparent overlaps. In contrast, Ethiopia and Kenya, covering a combined area of over 1.6 million square kilometers, share closely related vegetation types. This ecological similarity, along with better documentation, likely explains the higher number of shared invasive species between them. Although Sudan’s land area is comparable to the combined size of the other countries, the number of reported invasive plant species may have been underestimated due to insufficient data and underreporting in the literature^32^. This implies the need for further assessment studies on invasive species across the region.

### Origin of the invasive plants

The native range of the majority of the invasive species documented in the region was found to be the neotropical biogeographic realm. This could probably be due to numerous reasons that could be attributed to the similarities in climatic conditions or human-induced factors. However, species-specific adaptive responses to heterogeneous environments also play a crucial role.

The Neotropical, African (Afrotropical), and Saharo-Arabian biogeographic realms manifested some climatic similarities especially due to the pronounced seasonality in rainfall, high temperatures, and prolonged dry periods, that have shaped their ecosystems^51^. Similarly, the African realm includes extensive arid regions such as the Horn of Africa biodiversity hotspot, which is also called the Arid Horn, reflecting a predominantly dry climate with seasonal variability^33^. Although the Neotropical realm is generally more humid and tropical, it also contains dry forest and savanna ecosystems that experience pronounced dry seasons, showing a climatic gradient that partially overlaps with arid conditions found in parts of the African and Saharo-Arabian realms. These realms thus share climatic features of seasonal rainfall variability and historical shifts between wetter and drier periods, influenced by monsoon dynamics and continental positioning, which have shaped their distinct but related floristic and faunal compositions^51^. This would help the invasive plant species to become successful once they reach the Horn of Africa.

The neotropics is also a region which is believed to host the largest species pool and richness^70^ and it is highly likely to inherently offer more potential invaders, simply because more species exist due to propagule pressure through global trade and human travel^71^. Once they reach the study area, their success might have been determined by the enemy release and novel biotic and abiotic advantages hypothesis^58,72^. Although comparative phenological studies are limited in the region, the invasive species might also have an advantage of growing when the native species slow down, or exploiting open canopy conditions created by disturbance. Furthermore, the study area is extremely degraded and deforested which leaves a large open niche for invaders, which aligns to the niche occupancy hypothesis. For example, in the lowland areas of the Horn of African countries, e.g. Ethiopia, *N. juliflora* invades the barelands easily. Despite its presence, however, it’s not a strong invader in the forests, shrublands or even grasslands dominated by native perennial grasses.

In response to the loss of native species caused by global change factors, humans may have attempted to compensate by introducing non-native species^22^, further entangling anthropogenic activities, global change, and invasive alien plant species in a complex web of interactions. The introduction of *Acacia saligna* (Fabaceae) and *Acacia mearnsii* (Fabaceae) is a typical example. These species were introduced to the region with the intention of stabilizing soil, supporting reforestation, and providing firewood. Ironically, despite being invasive in many parts of the world, including the study area, they continue to be planted in Ethiopia^73^. The international trade of goods and living organisms is also becoming the leading driver of species introductions and is expected to increase as global reliance on trade networks grows, particularly in sectors such as horticulture, urban green spaces, biofuels, and agriculture^55^.

### Invasive species occurrences along an elevation gradient

The invasive plant occurrence points revealed that the invasive species are predominantly distributed in the lower elevations although the middle elevation range is the peak. This is congruent with local and global studies^21,27,28,74^. The most possible reason is often explained by directional ecological filtering, where non-native species are typically introduced at lower elevations due to higher anthropogenic propagule pressure, and only those with broad climatic tolerances or pre-adaptations are able to successfully spread upwards^27,29,30^. In the present study, there are two potential reasons for the high invasive species occurrences at lower elevations than higher elevation.

The availability of large empty niche especially in the semi-desert and desert ecosystems in most of the target countries. A significant portion of the reported invasive plant species are distributed in these ecosystems. For example, the species with a large number of occurrence points in the study area, *N. juliflora*, is found in these ecosystems at lower elevation range^8^. This supports the vacant niche occupancy hypothesis^75,76^.

Another possible explanation is that lower elevation areas experience more intense anthropogenic disturbances such as agriculture, deforestation, overgrazing, and urbanization. Besides the deliberate introduction of non-native plants for purposes like ornamental use and urban forestry, unintentional introduction and dispersal through road networks are also highly probable. This perspective is supported by recent studies indicating that human activities largely determine the abundance of invasive species^23,77^, although da Rosa et al.^23^ argue that elevation itself plays a minimal role in shaping invasive species distribution. However, anthropogenic activities are closely linked with elevation because, for example, plants grown at lower elevations cannot thrive at higher, colder altitudes, and cities are typically established in lower elevation zones. Thus, the interaction between human disturbances and elevation influences invasive species patterns^27,78^.

However, global change factors, especially the interconnected impacts of climate change and land use/land cover change, may eventually affect even high-elevation areas. This implies that although invasive plant species are currently most common at low to mid elevations, this distribution could shift in the future^79^.

### Road proximity and invasive plant occurrence

The abundance of invasive plant species decreased with increasing distance from the road, with nearly 75% of occurrence points recorded within one kilometer. Similar patterns have been observed globally^24,26,67,80^. While various explanations are possible, we discuss the following three scenarios.

One possible explanation is that road networks may act as corridors for the transport and spread of invasive plant species^80,81^. In the study area, cross-country road networks in particular appear to facilitate the introduction and dispersion of invasive plants. A well-documented example is the unintentional introduction of *P. hysterophorus* into Ethiopia via trucks and trains traveling along the Dire Dawa road corridor, which links the country to Djibouti and Somalia^81^. Given that roads are the primary terrestrial infrastructure linking the target countries, the extent of the issue may be more beyond the current reality. Transnational research in Europe also proved that roadsides and rail networks served as colonization corridors for invasive plant species^82^.

A second important factor may be that road networks create habitats with significantly reduced competition, thereby facilitating the spread and establishment of invasive plant species^83,84^. Based on previous research experience, *Nicotiana glauca* (Solanaceae) is a prime example of a species that takes advantage of these conditions, often rapidly colonizing newly constructed roadsides.

A third possible explanation could be the ease of access for data collectors and researchers, which likely led to a higher record of data points near roads. In regions like the study area, where infrastructure is limited and resources are scarce, researchers may naturally prioritize easily accessible locations. Furthermore, road networks along mountains are poorly developed, which makes them hardly accessible for researchers. While this may introduce a certain degree of bias, it reflects the practical challenges faced in the field.

### Distribution of invasive plants across landcover types

Land use and land cover change significantly influence the distribution and abundance of invasive plant species, often acting as primary drivers of their spread and establishment. This has been extensively studied^1,2,22,23,54,57^. The implication is that the more the human activities are in a certain area, the more invasive species they exist there. The present study also reported that the land cover types that are highly affected by human activities i.e., cultivated land, urban and built areas were the highest records observed. This finding is in line with reports elsewhere^13,23,55,80,83^. This is attributed to the fact that roads, tracks, trails, irrigation, and other infrastructure are consistently identified as primary pathways and hotspots for the introduction and rapid spread of non-native species, acting as dispersal corridors and providing disturbed, suitable habitats^23,80,82^. The shrublands in the study area are also the outcome of millennial land use and land cover changes^85^.

On the other hand, the less disturbed areas, particularly the forests, hosted relatively lower records. This is also supported by both experimental studies and surveys^86,87^. This could be fundamentally due to two reasons i.e., either the invasive plant species are not reaching the forests or they are not able to establish once they reach there because of the biotic and abiotic conditions there. In support of this argument Yang et al.^88^ also concluded that terrestrial invasive herbs and shrubs do not pose a serious threat to native species in the closed-canopy tropical forests, which is linked to habitat resilience.

## Conclusions and policy implication

The number of invasive plant species reported in the study area is notably high. Among the countries in the Horn of Africa, Kenya and Ethiopia were found to host the highest number of invasive plant species. This could be due to to their ecological similarities, closer geographical proximity, and a relatively higher number of studies conducted in these countries, which has led to better documentation.

The distribution of invasive species along the elevation gradient showed a clear decline with increasing altitude. Most records were concentrated at lower elevations, likely due to the availability of unoccupied ecological niches, particularly in semi-desert and desert ecosystems, as well as greater human disturbances such as agriculture, deforestation, overgrazing, and urbanization, which are common at lower altitudes.

Additionally, the data revealed a negative correlation between the abundance of invasive species and the distance from roads: as distance from roads increases, the number of recorded invasive plant species decreases. This pattern suggests that roads may act as dispersal corridors, facilitate the establishment of invasive species by creating disturbed habitats with reduced competition, or simply reflect a sampling bias due to easier access for researchers and data collectors.

The study identified four major limitations. First, the protocols used to assess the impact and classify plant species as invasive were often not reported. Commonly accepted tools such as EICAT, GISS, HARMONIA+, or NNRA were rarely cited, if at all. Second, many studies assume that if a plant species is invasive in one area, it is invasive wherever it is recorded, an assumption that may stem from the absence of standardized assessments. For instance, if an initial study incorrectly classified a species without a formal approach, subsequent research might adopt that classification without validation, which is a ‘*snowball effect*’. The third reason is the paucity of data on invasive plant species, as there is extremely limited literature available for the countries other than Kenya and Ethiopia. A fourth possible reason is the inconsistent understanding and unclear definitions of terms such as invasive species, exotic/alien species, and established/naturalized alien plants, which can lead to misinterpretation of what constitutes an invasive plant species, potentially inflating or underestimating the extent of invasion.

These four limitations likely affect the accuracy of the reported number of invasive plant species in the region. The actual number may either be overestimated or underestimated.

We forward the following recommendations:

- Development of expert and region based invasive plant species impact assessment protocol or adopting existing protocols such as EICAT and assess invasiveness and impact of the uncertain invasive plant species.
- Involve citizen science in invasive plant species research especially in the countries where literature is extremely limited.
- Develop a collaborative strategic invasive plant species management framework across the Horn of African countries and work accordingly.

## Supplementary Information

Below is the link to the electronic supplementary material.

Supplementary Material 1.

Supplementary Material 2.

Supplementary Material 3.

## Data Availability

Data generated or analyzed during this study are included in this manuscript and its supplementary information files.
